# Evaluation of Mosquito Attractant Candidates Using a High-Throughput Screening System for *Aedes aegypti* (L.)*, Culex quinquefasciatus* Say. and *Anopheles minimus* Theobald (Diptera: Culicidae)

**DOI:** 10.3390/insects12060528

**Published:** 2021-06-06

**Authors:** Dae-Yun Kim, Theerachart Leepasert, Michael J. Bangs, Theeraphap Chareonviriyaphap

**Affiliations:** 1 Department of Entomology, Faculty of Agriculture, Kasetsart University, Bangkok 10900, Thailand; daeyun.k@ku.th; 2 Department of Chemistry, Faculty of Science, Kasetsart University, Bangkok 10900, Thailand; fscitcl@ku.ac.th (T.L.); bangs_michael@yahoo.com (M.J.B.)

**Keywords:** attractant, BG-lure, high-throughput screening system assay, KU-lure, olfactometer, *Aedes aegypti*, *Culex quinquefasciatus*, *Anopheles minimus*, diffusion assay

## Abstract

**Simple Summary:**

Trapping mosquitoes can enhance its capture rate by adding attractants such as carbon dioxide or human hosts’ odor-mimicking synthetic blends. Various olfactometers exist to test mosquitos’ behavior, but high-throughput screening system (HITSS)—one of the diffusion assays—has not been applied to developing lures. In this study, six different newly prepared chemical lure candidates (Kasetsart University (KU)-lures) were tested for diurnal *Aedes aegypti*, nocturnal *Culex quinquefasciatus* and nocturnal *Anopheles minimus*, using the HITSS assay. Results showed species-specific different lure preferences; the diurnal species were attracted to KU-lure #1 (29.7%), while both of the nocturnal species preferred KU-lure #6 (68.3% and 74.3% for *Cx. quinquefasciatus* and *An. minimus*, respectively). In addition, the selected lure candidates clearly demonstrated dose-dependent reversal responses for each *Ae. aegypti* and *Cx. quinquefasciatus*. Our results indicate that the HITSS assay distinguishes potential species-specific lure candidates. In addition, the HITSS assay was equally effective in determining the host-seeking behavior in pyrethroid-resistant and -susceptible strains. Further studies are needed to determine the accuracy of the HITSS assay in large-scale semi-field screen house tests using commercial traps.

**Abstract:**

Several types of olfactometers have been used to evaluate mosquito responses to agents that mimic natural volatiles that repel or attract. The Y-tube olfactometer has been widely used to study repellents and attractants, while the high-throughput screening system assay has only been used to study repellents. Whether the high-throughput screening system assay is suitable for evaluating attractants is unknown. We evaluated the responses to four lactic-acid-based mixtures and two non-lactic-acid-based chemical lure candidates using the high-throughput screening system (HITSS) for three mosquito species (laboratory strains and field populations of both *Aedes aegypti* (L.) and *Culex quinquefasciatus* Say.; laboratory strain of *Anopheles minimus* Theobald) under laboratory-controlled conditions. HITSS assay results showed that KU-lure #1 elicited the greatest percent attraction for pyrethroid-resistant and -susceptible *Ae. aegypti.* KU-lure #6 elicited the strongest attractive response for pyrethroid-susceptible and -resistant *Cx. quinquefasciatus* and pyrethroid-susceptible *An. minimus*. The response to the lures from each species was independent of the pyrethroid susceptibility status (*Ae. aegypti*, *p* = 0.825; *Cx. quinquefasciatus*, *p =* 0.056). However, a significant difference in attraction to KU-lure #6 was observed between diurnal and nocturnal mosquitoes (*Cx. quinquefasciatus* vs. *Ae. aegypti*, *p* = 0.014; *An. minimus* vs. *Ae. aegypti*, *p* = 0.001). The laboratory-level HITSS assay effectively selects potential lure candidates. Because the host-seeking behavior differs between mosquito species, further studies are needed to develop species-specific attractants. Additional studies in semi-field screen houses using commercial traps are necessary to evaluate the accuracy of these laboratory assay results.

## 1. Introduction

Mosquito traps with electric fans have been developed for use in mosquito surveillance and management [1,2,3]. These devices use a light source or carbon dioxide to attract the mosquitoes. However, the female mosquito’s host-seeking behavior involves more than the detection of carbon dioxide [4,5,6]. To increase the catch rate, artificial attractants that mimic the host odor have been investigated for their ability to lure mosquitoes to the device [7,8]. A variety of components of host odor have been evaluated and analyzed by chromatography and electroantennography [9,10]. Most of the aromatic molecules known to promote responses in mosquitoes and other hematophagous arthropods are either short chain carboxylic acids, aldehydes, or low molecular weight nitrogenous compounds such as ammonia [11]. Host chemicals, including L-lactic acid, ammonia, octenol (1-octen-3-ol), indole, nonanal (nonanaldehyde); components of host red blood cells; and select amino acids may serve as attractant cues at a close distance to the host, together with the host’s body heat (infrared spectrum) and surface moisture [12,13,14].

The response of host-seeking female mosquitoes to attractants has been studied using olfactometers [9,15,16,17,18,19,20,21]. A variety of olfactometers have been developed since the early 1990s [22,23,24]. The Y-tube olfactometer, one of the most widely used tools, provides airflow as the female mosquitoes fly upwind along an attractant gradient toward the source [9,17,25]. However, evidence suggests that the artificial air flow in testing devices, unlike natural conditions, may create confusion, thereby affecting the mosquito’s host-seeking behavior [15]. How the air plume influences the host-seeking mechanism in mosquitoes has been described in detail [10]. The World Health Organization (WHO) [21] recommends the high-throughput screening system (HITSS) as an alternative method for testing attractants. This simple diffusion assay does not involve airflow and thus is free from artificial air biases. This method can also be used in a wider range of settings, as it does not require a sophisticated laboratory [20]. The HITSS comprises three protocols for testing synthetic or natural repellent active ingredients: the toxicity assay, contact irritancy assay, and the spatial repellency assay [26]. However, the HITSS protocols were designed for identifying repellents, not attractants. Recently, Kim et al. [27] revealed that the HITSS assay successfully optimizes Biogent (BG)-lure^TM^ against *Aedes aegypti* and *Culex quinquefasciatus*, but whether the HITSS assay is suitable for screening new synthetic lures is unknown. In addition, behavioral studies using olfactometers against local mosquito populations are necessary because there is geographical variation [28] which correlates with pesticide resistance and olfactory responses [29].

To answer this question, we evaluated the function of an HITSS device for assessing multiple lure candidates against susceptible and resistant strains of both *Ae. aegypti* and *Cx. quinquefasciatus*. To expand capability of the HITSS assay, we added a malaria vector: *Anopheles minimus* laboratory strain. The HITSS screening assay results were then used to determine the differences in host-seeking behavior within (pesticide-resistant versus -susceptible) and between species (diurnal versus nocturnal) and strains.

## 2. Materials and Methods

### 2.1. Mosquito Strains

Laboratory strains. *Aedes aegypti* was provided in 2001 by the U.S. Department of Agriculture, Gainesville, FL and has been continually maintained at the Department of Entomology, Faculty of Agriculture, Kasetsart University, Bangkok, Thailand under laboratory-controlled rearing conditions. *Culex quinquefasciatus* was obtained from the National Institute of Health, Department of Medical Sciences, Ministry of Public Health, Nonthaburi, Thailand. The colony has been maintained at the Department of Entomology, Faculty of Agriculture, Kasetsart University since 2015. *Anopheles minimus* colony has been maintained since 1998, with specimens provided by the Malaria Division, Department of Communicable Disease Control, Ministry of Public Health, Nonthaburi, Thailand. All colonies are completely susceptible to all insecticides.

Field strains. *Aedes aegypti* larvae and pupae were collected from natural breeding habitats (containers) at Pu Tuey Village, Sai Yok District, Kanchanaburi Province (14°17′ N, 99°11′ E). *Culex quinquefasciatus* larvae and pupae were collected from sewage (local restaurant) at Thawi Watthana in Bangkok, Thailand (13°77′ N, 100°34′ E). All larvae and pupae were immediately transferred to the Department of Entomology, Faculty of Agriculture, Kasetsart University in Bangkok. F2 adults of each species were tested for pesticide susceptibility according to the WHO bioassay protocol.

### 2.2. Mosquito Rearing Techniques

*Aedes aegypti*, *Cx. quinquefasciatus* and *An. minimus* were uniformly reared under insectary-controlled conditions (25 °C ± 5 °C, 80% ± 10% RH, and 12:12 light:dark photoperiod) at the Department of Entomology, Faculty of Agriculture, Kasetsart University, following previously described methods [30,31,32].

*Aedes aegypti*. Adults were provided with cotton pads soaked with 10% sugar solution on the first day of emergence and were maintained in separate screen cages. The naturally mated female mosquitoes were permitted to feed on blood through an artificial membrane feeding system on day 3 post-emergence. Two days after blood feeding, 10 cm diameter oviposition dishes containing moist filter paper were placed in the cages for egg deposition. Eggs were dried at room temperature for two days before being immersed in water in individual hatching trays. At two days post-hatch, 200–250 larvae were transferred to individual plastic rearing trays (30 cm [L] × 20 cm [W] × 5 cm [H]) containing clean water. Larvae were fed once daily using a commercially sourced protein mixture as larval food (Optimum^TM^ Nishikigoi Carp Fish, Perfect Companion Group Co., Ltd., Samutprakarn, Thailand). Pupae were transferred daily from larval trays to emergence cups and placed directly into mosquito-proof screen cages covered with steel mesh (30 cm [L] × 30 cm [W] × 30 cm [H]). Adults were maintained in screen cages with a 10% sucrose solution before assays.

*Culex quinquefasciatus*. Eggs were laid in rafts directly on the water surface, with 250–300 eggs per raft. The intact egg rafts were gently transferred with a wooden applicator stick and placed on the water surface of similar larval trays. Larvae were fed the same as *Ae. aegypti*. Pupae were transferred daily from larval trays to emergence cups and placed directly into mosquito-proof screen cages covered with steel mesh (30 cm [L] × 30 cm [W] × 30 cm [H]). Non–blood-fed female mosquitoes (3–5 days old) were starved (provided only with water) for 24 h before testing.

*Anopheles minimus*. Larvae were fed powdered TetraMin^®^ tropical fish food daily. Pupae were collected and transferred to a screen cage where they were enclosed as adults. Adults were maintained in screen cages with a 10% sucrose solution as an energy source. All mosquito cages were covered with damp towels to retain moisture. Three to five-day-old non-blood-fed female mosquitoes were starved (provided only with water) for 24 h before testing.

### 2.3. Experimental Assays

Biogents (BG)-lure. BG-lure (Biogents, Regensburg, Germany) purchased from BioQuip (Rancho Dominguez, CA, USA) contains a mixture of at least three active ingredients: 20–40% L-(+)-lactic acid, 20–40% ammonium hydrogen carbonate, 5–10% hexanoic acid, and other inert ingredients.

Kasetsart University (KU)-lure candidates. Six KU-lure candidates with different chemical compositions were investigated. Four mixtures contained L-lactic acid (Tokyo chemical industry Co., LTD., Tokyo, Japan) and isovaleric acid (Sigma-Aldrich, St. Louis, MO, USA) in various proportions (% *w*/*v*). For example, KU-lures #1 and #6 consisted of the same ingredients, but the latter had lower concentrations. KU-lures #4 and #5 were based on the same concentration of lactic acid, but the former was mixed with two more compounds: ammonium hydroxide and myristic acid. KU-lures #2 and #3 are single compounds of isoamyl alcohol and octanol, respectively (Table 1).

Based on the results of tests to determine the optimal dose, we used KU-lure #1 for *Ae. aegypti* and KU-lure #6 for *Cx. quinquefasciatus* for further studies.

### 2.4. High-Throughput Screening System

**Figure 1:**
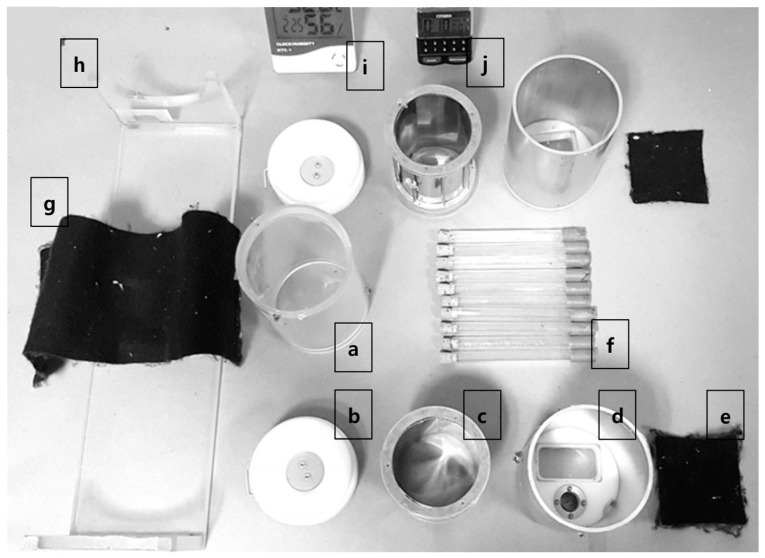
Assembly parts of high-throughput screening system: (**a**) middle Plexiglas cylinder; (**b**) funnel cap for connecting chambers and opening/closing butterfly doors; (**c**) inner metal drum for holding filter paper; (**d**) outer metal chamber; (**e**) fabric window cover of the outer metal chamber; (**f**) mosquito holding tubes; (**g**) fabric to cover the transparent middle cylinder; (**h**) cradle to place the HITSS assembly; (**i**) hygrometer; (**j**) timer.

The HITSS diffusion assay for evaluating repellents [26] consists of three hollow cylinders, two metal and one acryl, linked by connecting doors (Figure 1). Between the two metal chambers is a transparent acrylic material (Plexiglas^®^) chamber [5 cm (D) × 10 cm (H)] assembled with butterfly-flap operated openings at each end. The mosquitoes were transferred into the middle chamber through a small hole (3.0 cm [D]) and could move freely from the middle chamber to access the two adjoining cylindrical chambers. One chamber contained the lure (2 cm × 2 cm filter paper impregnated with the test agent), while the other was the control (untreated filter paper) (see graphical abstract for assembled image).

Pyrethroid-susceptible and -resistant strains of both *Ae. aegypti* and *Cx. quinquefasciatus* were tested and compared within and between species. A susceptible strain of *An. minimus* was also tested. A group of 10 healthy female mosquitoes were collected using an aspirator and placed in a clean plastic mesh-covered cup and monitored for an hour. A total of 20 healthy mosquitoes were transferred using an aspirator from two cups into a single holding tube. In total, nine tubes were prepared for each assay (nine replicates in total) (Figure 1). The 20 mosquitoes were carefully introduced into the central HITSS through the 3 cm diameter hole by blowing, first checking to ensure the butterfly-flap doors were in the closed position. The transparent middle cylinder was immediately covered by a dark fabric to avoid phototaxic responses.

The mosquitoes in the middle chamber were allowed a 30 s period of adjustment before opening the butterfly-flap doors at each end. Once the doors were opened, the mosquitoes were able to fly freely between the three linked cylinders. After 10 min, the butterfly doors were closed, and the number of mosquitoes in each cylinder was counted. All replicates were conducted under standard conditions (25 °C ± 2 °C; 60% ± 5% RH).

### 2.5. Statistical Analysis

Attraction was measured as the difference between the number of mosquitoes found in each chamber, as a proportion of the total number of mosquitoes in both. To compare the attraction levels between candidates, the proportion of mosquitoes in the untreated and treated chambers was calculated using the following equation [33]:

(1) $$\mathrm{Percent}\;\mathrm{attraction}\;=\;\frac{\mathrm{number}\;\mathrm{of}\;\mathrm{mosquitoes}\;\mathrm{in}\;\mathrm{treated}\;\mathrm{chamber}\;-\;\mathrm{number}\;\mathrm{of}\;\mathrm{mosquitoes}\;\mathrm{in}\;\mathrm{untreated}\;\mathrm{chamber}}{\mathrm{number}\;\mathrm{of}\;\mathrm{mosquitoes}\;\mathrm{in}\;\mathrm{treated}\;\mathrm{chamber}\;+\;\mathrm{number}\;\mathrm{of}\;\mathrm{mosquitoes}\;\mathrm{in}\;\mathrm{untreated}\;\mathrm{chamber}}\;\times \;100$$

where 100% indicates complete attractant, 0% indicates no response, and −100% indicates complete repellent. This equation is derived from the preference index described in previous studies [34] and indicates the response of mosquitoes to attractants using a Y-tube olfactometer. The spatial activity index was originally used for data from the Spatial Repellency Assay [26] using HITSS. The Wilcoxon signed rank test was used to determine the significance of differences between mosquitoes in treated and untreated chambers (*p* < 0.05). Data are presented as the mean ± standard deviation (SD) of the percent attraction between different KU-lure candidates as determined using the Kruskal–Wallis *H* test for multiple comparisons (*p* < 0.05). To determine the relationship between pesticide resistance and host-seeking behavior, percent attraction was compared between susceptible and resistant strains of *Ae. aegypti* and *Cx. quinquefasciatus* using the Mann–Whitney *U* test. The Kruskal–Wallis *H* test for multiple comparisons was used to compare species-specific lure preferences between three species of mosquitoes: *Ae. aegypti*, *Cx. quinquefasciatus*, and *An. minimus* (all laboratory strains). All statistical analyses were performed using SPSS version 20 (IBM Corp., Armonk, NY, USA). Significance was set at 5% (*p* < 0.05).

## 3. Results

### 3.1. WHO Susceptibility Bioassay

Results confirmed that permethrin resistance was very high in *Ae. aegypti* (6% mortality at a discriminating dose for *Ae. aegypti*, 0.25% permethrin) and moderate in *Cx. quinquefasciatus* (60% at 0.75% permethrin). In contrast, all the susceptible strains showed 100% mortality in response to permethrin exposure.

### 3.2. HITSS Assay

Results of preliminary studies to optimize the lure dose are shown in Table 2. For the diurnal *Ae. aegypti* susceptible strain, significantly more mosquitoes were attracted to the chamber containing KU-lure #1 (0.01 g) than the untreated chambers (*p* = 0.024). The use of lower or higher doses of KU-lure #1 (0.001, 0.005, and 0.02 g) resulted in no significant difference between the treated and untreated chambers. For the nocturnal species *Cx. quinquefasciatus*, 0.005 and 0.02 g of KU-lure #6 attracted significantly more mosquitoes to the lure-treated chamber (*p =* 0.028 and 0.027, respectively). Because the percent attraction elicited by 0.01 g of KU-lure #6 did not differ significantly from that by 0.005 or 0.02 g (*p* = 0.965 and 1.000, respectively), we selected 0.01 g as the discriminating dose for all strains and chemicals tested in this study (Table 2 and Table 3).

Overall, the results indicate that the responses of mosquitoes to the chemical lures tested in the HITSS assay system vary depending on the strain. Data regarding the mean number of mosquitoes attracted or repelled by each KU-lure candidate (including negative and positive controls) are shown in Table 3. Responses to the standard BG-lure between treated and untreated chambers differed significantly, regardless of strain (*p* < 0.05).

The responses of each mosquito strain differed significantly between lure candidates. For example, the pyrethroid-susceptible strain of *Ae. aegypti* exhibited significantly greater attraction to the KU-lure #1 treated chamber than the untreated chamber (*p* = 0.024). The susceptible and resistant strains of *Cx. quinquefasciatus* exhibited greater attraction to the KU-lure #6 treated chamber than the untreated chamber (*p* = 0.007 and 0.017, respectively). The susceptible strain of *An. minimus* exhibited significantly greater attraction to the KU-lure #6 treated chamber than the untreated chamber (*p* = 0.014).

The attraction level of three mosquito species to the different lure candidates is reported as percent attraction. Overall, the standard BG-lure elicited the greatest positive response for all mosquito strains tested. For example, the mean percent attraction for susceptible *Cx. quinquefasciatus* and susceptible *An. minimus* was 68.3 ± 25.4 and 66.7 ± 45.8, respectively. A high percent attraction to the BG-lure was also seen for the two strains of *Ae. aegypti* (susceptible, 53.5 ± 31.8; resistant, 37.4 ± 35.9).

**Figure 2:**
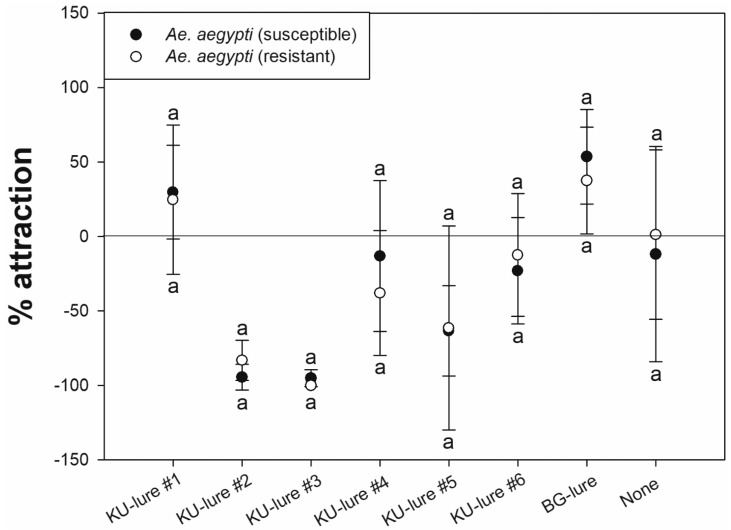
Percent attraction to each KU-lure (0.01 g) and BG-lure positive control (0.05 g) as measured by HITSS. Results are compared between pyrethroid-susceptible and -resistant strains of *Aedes aegypti*. A positive value indicates attraction to the treated chamber, while a negative value indicates repulsion from the treated chamber (more mosquitoes in the untreated chamber). For all lures, no significant difference in percent attraction was observed between mosquito strains (Mann–Whitney *U* test, *p* < 0.05).

KU-lure #1 elicited the greatest percent attraction for both susceptible and resistant *Ae. aegypti* (29.7 ± 31.4 and 24.6 ± 50.1, respectively). The level of attraction of both *Ae. aegypti* strains to KU-lure #1 did not differ significantly from that toward the BG-lure positive control (*p* = 0.155 and 0.721, respectively). All other KU-lures showed negative values for both strains of *Ae. aegypti*, indicating repellent activity (Table 3, Figure 2). For example, the Mann–Whitney *U* test showed no significant differences between the two strains of *Ae. aegypti* in the degree of attraction to the lures (KU-lure #1, *p* = 0.825; BG-lure, *p* = 0.417) or repellency or avoidance of the lures (KU-lure #2, *p* = 0.063; KU-lure #3, *p* = 0.113; KU-lure #4, *p* = 0.387; KU-lure #5, *p* = 0.605; KU-lure #6, *p =* 0.297) (Figure 2).

*Culex quinquefasciatus* showed the highest percent attraction to KU-lure #6. Furthermore, the level of attraction to KU-lure #6 did not differ significantly from that toward the BG-lure positive control for both susceptible and resistant *Cx. quinquefasciatus* (*p* = 0.258 and 0.730, respectively) (Table 3).

**Figure 3:**
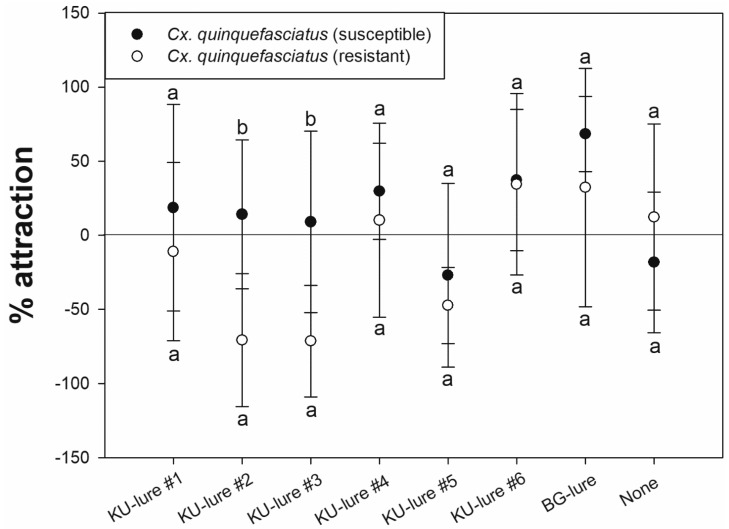
Percent attraction to KU-lures (0.01 g) and the BG-lure positive control (0.05 g) as determined by HITSS assay. Results were compared between pyrethroid-susceptible and -resistant strains of *Culex quinquefasciatus*. A positive value indicates attraction to the treated chamber, while a negative value indicates repulsion from the treated chamber (more mosquitoes in the untreated chamber). For all lures, except KU-lures #2 and #3, the Mann–Whitney *U* test showed no significant difference between mosquito strains (*p* < 0.05).

Responses to the four KU-lure mixtures did not differ significantly between susceptible and resistant *Cx. quinquefasciatus* (KU-lure #1, *p* = 0.489; KU-lure #4, *p* = 0.605; KU-lure #5, *p* = 0.448; KU-lure #6, *p* = 1.000). However, the two single compounds, KU-lure #2 and #3, resulted in significantly different responses between strains (*p* = 0.002 and 0.008, respectively) (Figure 3).

For the *An. minimus* laboratory strain, the highest percent attraction to KU-lure #6 was observed and it was not significantly different to BG-lure (74.3 ± 42.0 and 66.7 ± 45.8, respectively, *p* = 0.730) (Table 3).

**Figure 4:**
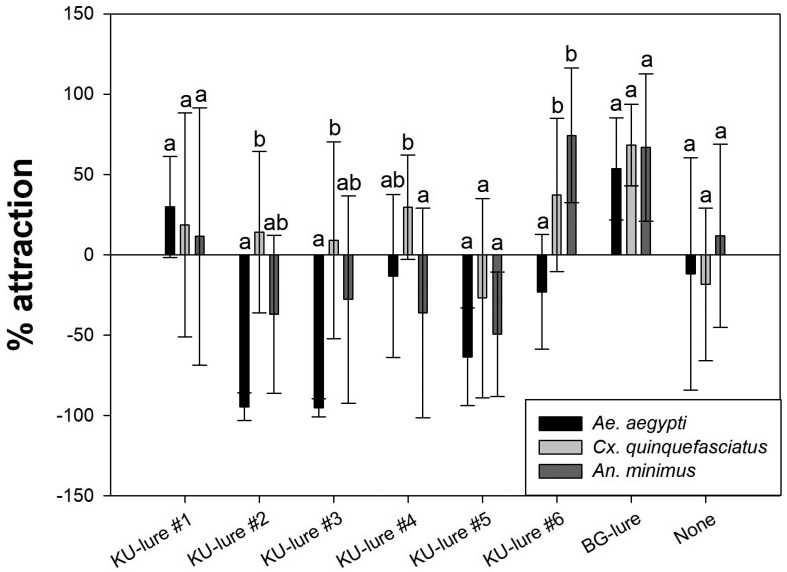
Percent attraction between susceptible strains of *Aedes aegypti*, *Culex quinquefasciatus*, and *Anopheles minimus*. Each KU-lure candidate tested with 0.01 g while BG-lure was 0.05 g. Different letters on bar indicate statistical significance with 95% confidence using Kruskal–Wallis *H* test for multiple comparisons (*p* < 0.05).

Comparison of the HITSS screening assay results between the susceptible strains of *Ae. aegypti, Cx. quinquefasciatus*, and *An. minimus* is shown in the bar graph in Figure 4. No significant difference in percent attraction was observed between the species toward any of the KU-lures except KU-lure #6, to which *Ae. aegypti* was significantly less attracted than both *Cx. quinquefasciatus* (*p =* 0.014) and *An. minimus* (*p* = 0.001).

## 4. Discussion

Using the high-throughput screening system (HITSS) assay, we could successfully optimize and screen the most effective lure candidates for each species of mosquitoes. In particular, KU-lures #1 and #6 were optimized, and the dose (0.01 g) successfully attracted *Ae. aegypti* and *Cx. quinquefasciatus*, respectively. The HITSS optimizing assay demonstrated a dose-dependent reversal response, which was observed in a previous study using BG-lure [27]. The species-specific differences reappeared in this study; the attractive dose range for *Cx. quinquefasciatus* (0.005 g to 0.020 g) was two times wider than that for *Ae. aegypti* (0.005 g to 0.010 g).

The KU-lures investigated in this study were not evaluated previously. The compounds and mixtures were chosen based on the results of attraction studies investigating L-lactic acid [12,35], 1-octen-3-ol [36], isovaleric acid (syn. 3-methyl butanoic acid) [37,38], isoamyl alcohol [39], myristic acid [40], and ammonium hydroxide [9]. For example, because female *Ae. aegypti* are reported to be attracted to L-lactic acid, ammonia, and fatty acid mixtures [9,41,42,43], four KU-lure mixtures contained L-lactic acid as the main compound; other chemicals such as isovaleric acid and/or octenol (KU-lures #1 and #6), myristic acid, and ammonium hydroxide (KU-lure #4) were added in a range of percentage compositions. However, L-lactic acid alone was not an attractant [17]. 

Isoamyl alcohol (100% *w*/*v*) generated by yeast fermentation of sucrose [39] was tested using the HITSS assay (KU-lure #2) in this study, but the single compound was not attractive for any mosquitoes tested. In addition, although Mathew et al. [40] reported that 1-octen-3-ol alone strongly attracted *Ae. aegypti*, from our results, we could not observe either *Ae. aegypti* and *Cx. quinquefasciatus* being attracted to the 100% *w*/*v* octenol (KU-lure #3).

The differences in attraction to lures between mosquito species reflect species-specific differences in their olfactory receptor neurons (ORNs) [44,45]. For example, comparison of brown (village) and black (forest) *Ae. aegypti* in Kenya revealed 14 genes in the village species related to the detection of human host cues that were absent in the forest species [46]. As the forest species moved to urban areas, they developed ORNs to enable the detection of humans. The *Ae. aegypti* odorant receptor AaegOr4 responds strongly to sulcatone (syn. 6-methyl-5-hepten-2-one) in human sweat. The ORNs are highly sensitive, detecting small amounts and low concentrations of their targets (sensitivity), and are selective for sensing different odorants (selectivity) [35,43,47].

Human odor generated from sweat and skin is attractive to a number of mosquito species, but the host odor composition determines its attractiveness to different mosquito species [48]. The mosquito response to these odors appears to be activated by carbon dioxide, which is universally emitted from vertebrates. In addition, carbon dioxide is responsible for increased flight activity and attraction of mosquitoes to host odors [1]. Studies suggest that *Cx. quinquefasciatus* is more strongly attracted to carbon dioxide than is *Ae. aegypti* [47,49]. It has been confirmed using HITSS assay that the nocturnal species were significantly more attracted than the diurnal species towards 1.0 g of dry ice (76% ± 26.3% vs. 12.2% ± 29.6%, *p* = 0.002) [27].

The HITSS device does not require electric power, and its compact metal body makes it easy to transport. This device was originally intended for mass screening of compounds for repellency and toxicity [26]. This study is the first to use HITSS to screen attractants.

In this study, the HITSS assay successfully identified the most strongly attractive lure for each mosquito species tested. For both diurnal and nocturnal species, lures containing mixtures of ingredients attracted more mosquitoes than did single-component lures. Animal skin surfaces carry thousands of compounds, but only a few function as kairomones [18]. Exhaled breath contains not only carbon dioxide but also heat and moisture, along with volatile organic compounds from blood such as acetone and butanone. Human sweat attracts significantly more mosquitoes than moisture [24]. Analysis of attractants in human sweat has shown that while lactic acid is the major attractant component, it does not attract mosquitoes alone [12]. The addition of carbon dioxide to lactic acid increases its attractant activity, and mixtures such as lactic acid + octenol + carbon dioxide are demonstrated efficacious attractants [19]. Electrophysiological studies have revealed the detailed composition of human sweat [50,51] and identified effective mixtures of several compounds that work synergistically in a dose-dependent and species-specific manner [9,51].

Our results show that the optimal composition of the KU-lures investigated here are species specific. For example, KU-lures #1 and #6 for each species contained the same chemicals (L-lactic acid, 1-octen-3-ol, and isovaleric acid), but in different ratios (10:2:4 vs. 2:0.25:0.5, respectively). A previous study showed the importance of composition and component ratios in lure mixtures [52]. Concentrations are critical, even for carbon dioxide, which repels female host-seeking mosquitoes at high concentrations [24,53].

Since octenol is a confirmed an efficacious attractant [54], we mixed 2% *w*/*v* of 1-octen-3-ol with a five-times higher amount of L-lactic acid (10% *w*/*v*) and a 100-times higher amount isovaleric acid (4% *w*/*v*) than in the KU-lure #4 to create KU-lure #1. Compared to KU-lure #4, KU-lure #1 was more attractive to susceptible and resistant *Ae. aegypti*, but less attractive to the two nocturnal species, *Cx. quinquefasciatus* and *An. minimus*. These results are consistent with a previous study reporting that mixtures of two or three kairomones had a synergistic effect on attractiveness to *Ae. aegypti* [51]. Volatiles from host skin bacteria are known to attract mosquitoes [55]. Such a compound is isovaleric acid (syn. 3-methyl butanoic acid), one of the short-chain volatile fatty acids that cause strong axillary malodor. The KU-lure blends (#1, #4, #5 and #6) contained lactic acid and isovaleric acid as two base attractants. Each of these lures attracted mosquitoes in a dose-dependent and species-specific manner.

Understanding the olfactory receptors and odor molecular responding mechanisms are essential to develop lures. Sensilla, the insect sensory organs located on the antennae, contain ORNs that respond to specific chemicals. To match these chemicals to their specific ORNs, electroantennography has been used [50]. Odorant receptors and their responses to chemicals were investigated [35], revealing that about 10% of over 3000 compounds in human sweat are involved in the host-seeking responses of *Ae. aegypti* [51]. This information might be useful for identifying new combinations of components for use in mosquito attractants. However, the optimal proportions and mixtures of such components remain unknown. More studies are needed to investigate a range of mixtures and concentrations of components, and the HITSS assay may be useful for such analysis.

As multiple vector species typically reside in a given location, a variety of blends of customized candidates should be tested and developed to attract female mosquitoes of each local species [28]. In addition, a previous study confirmed reduced olfactory sensitivities from the pyrethroid-resistant strain of *Ae. aegypti* towards both repellents and attractants [29]. In this study, however, overall results do not represent the correlation. For example, no significantly different responses between susceptible and resistant *Ae. aegypti* were observed from any of the KU-lures tested (Figure 2). Subsequently, the susceptible and resistant strains of *Cx. quinquefasciatus* did not respond significantly differently to the KU-lure mixtures, as opposed to the KU-lures that were 100% *w*/*v* isoamyl alcohol or octenol (Figure 3). Both single compounds with high concentration strongly repelled or not attracted *Ae. aegypti, Cx. quinquefasciatus* and *An. minimus* (Figure 4). Although the pyrethroid-susceptible strain of *Cx. quinquefasciatus* demonstrated a significantly higher percent attraction compared to the pyrethroid-resistant strain towards KU-lures #2 and #3, the former strain was counted as not significantly different between treated and untreated chambers (Table 3). It was not uncommon to observe knockdown mosquitoes in the HITSS assay with high concentrations of the lure candidates. Previously, 10 g of BG-lure caused not only strong repellency, but also knockdown regardless of phenotypes in the HITSS assay [27]. So, the optimal doses or concentrations are critical to develop appropriate lures using the HITSS assay.

While the HITSS assay must be conducted in a laboratory, it can be used for pre-screening to reduce the time and effort required at the semi-field level using traps and the semi-field screen house (SFS) assay. The SFS assay yields information for determining the optimal amounts of an agent to achieve a suitable preference index. The best candidates optimized under semi-field conditions are then tested in field trials using different types of traps to determine the efficacy of the agent. This process will lead to improvements in candidate lures. A better understanding of the mechanisms underlying the attraction of mosquitoes toward human hosts is important for designing environmentally friendly attractant traps that are effective with less insecticide, in order to control vector populations and arthropod-borne disease transmission.

## 5. Conclusions

This laboratory-scale study of chemical lures using an HITSS assay establishes that the simple olfactometer originally designed for evaluating repellents can be applied to testing attractants. Our results indicate that the HITSS assay distinguishes potential species-specific lure candidates. In addition, the HITSS assay was equally effective in determining the host-seeking behavior in pyrethroid-resistant and -susceptible strains. Further studies are needed to determine the accuracy of the HITSS assay in large-scale SFS house tests using commercial traps.

## Figures and Tables

**Table 1:** Chemical composition of KU-lure candidates.

Compounds	KU-Lure # 1	KU-Lure # 2	KU-Lure # 3	KU-Lure # 4	KU-Lure # 5	KU-Lure # 6
Lactic acid	10% *w*/*v*	N/A	N/A	2% *w*/*v*	2% *w*/*v*	2% *w*/*v*
Octenol	2% *w*/*v*	N/A	100% *w*/*v*	N/A	N/A	0.25% *w*/*v*
Isovaleric acid	4% *w*/*v*	N/A	N/A	4% *w*/*v*	0.02% *w*/*v*	0.5% *w*/*v*
Isoamyl alcohol	NA	100% *w/v*	NA	NA	NA	NA
Myristic acid	NA	NA	NA	0.0025% *w*/*v*	NA	NA
Ammonium hydroxide	NA	NA	NA	2.5% *w*/*v*	NA	NA

NA, not applicable. Distilled water used as solvent for mixtures.

**Table 2:** Number of *Aedes aegypti* and *Culex quinquefasciatus* mosquitoes attracted to different amounts of each lure to determine the discriminating dose.

Species	Lure	Amount (g)	Number of Mosquitoes in Each HITSS Chamber (Mean ± SD)		*p* *	Percent Attraction ** (Mean ± SD)
			Untreated	Treated		
*Aedes aegypti* (Susceptible)	KU #1	0.000	2.2 ± 1.7	1.3 ± 1.1	0.203	−11.9 ± 72.2 ^c^
		0.001	1.0 ± 0.9	1.0 ± 1.1	0.952	−18.5 ± 93.0 ^c^
		0.005	2.1 ± 2.6	2.9 ± 1.7	0.339	32.9 ± 60.4 ^a^
		0.010	3.1 ± 1.5	6.0 ± 2.4	0.024 ^†^	29.7 ± 31.4 ^a^
		0.020	2.9 ± 1.4	3.6 ± 2.0	0.497	6.7 ± 45.7 ^b^
*Culex quinquefasciatus* (Susceptible)	KU #6	0.000	3.6 ± 2.7	3.7 ± 3.2	1.000	−18.4 ± 47.4 ^c^
		0.001	2.9 ± 2.7	2.4 ± 1.0	0.550	19.7 ± 65.9 ^b^
		0.005	3.2 ± 1.8	7.7 ± 3.2	0.028 ^†^	36.6 ± 38.0 ^a^
		0.010	3.2 ± 2.4	6.8 ± 2.7	0.066	37.2 ± 47.6 ^a^
		0.020	2.6 ± 1.9	5.3 ± 2.9	0.027 ^†^	41.7 ± 38.9 ^a^

* Wilcoxon signed rank test (*p* < 0.05). ^†^ Significantly more mosquitoes in the treated HITSS chamber. ** Percent attraction = (# mosquitoes in treated − # mosquitoes in untreated)/(# mosquitoes in treated + # mosquitoes in untreated) × 100. Different letters in columns indicate significant differences between species-specific doses using Kruskal–Wallis *H* test for multiple comparisons (*p* < 0.05).

**Table 3:** Mean number of mosquitoes attracted or repelled by the KU-lures.

Species	Lure #	Number of Mosquitoes in Each HITSS Chamber (Mean ± SD)		*p* *	Percent Attraction ** (Mean ± SD)
		Untreated	Treated		
*Aedes aegypti* (Susceptible)	KU #1	3.1 ± 1.5	6.0 ± 2.4	0.024 ^†^	29.7 ± 31.4 ^a^
	KU #2	14.9 ± 2.0	0.4 ± 0.7	0.007	−94.6 ± 8.6 ^d^
	KU #3	14.6 ± 3.9	0.4 ± 0.5	0.007	−95.3 ± 5.6 ^d^
	KU #4	2.3 ± 1.4	2.0 ± 1.6	0.599	−13.3 ± 50.6 ^b^
	KU #5	4.3 ± 2.1	1.2 ± 1.3	0.007	−63.5 ± 30.4 ^c^
	KU #6	3.1 ± 0.8	2.2 ± 1.1	0.066	−23.1 ± 35.6 ^b^
	BG-lure	1.9 ± 1.5	6.4 ± 2.7	0.007 ^†^	53.5 ± 31.8 ^a^
	None	2.2 ± 1.7	1.3 ± 1.1	0.203	−11.9 ± 72.2 ^b^
*Aedes aegypti* (Resistant)	KU #1	2.7 ± 1.8	4.3 ± 2.1	0.151	24.6 ± 50.1 ^a^
	KU #2	12.7 ± 3.0	1.1 ± 0.9	0.007	−83.2 ± 13.5 ^d^
	KU #3	16.8 ± 1.8	0.0 ± 0.0	0.007	−100.0 ± 0.0 ^d^
	KU #4	2.2 ± 1.1	1.1 ± 0.9	0.041	−38.1 ± 42.0 ^c^
	KU #5	3.2 ± 2.7	0.4 ± 0.7	0.019	−61.5 ± 68.5 ^cd^
	KU #6	2.8 ± 0.7	2.6 ± 1.3	0.581	−12.5 ± 41.1 ^bc^
	BG-lure	1.4 ± 0.7	3.2 ± 1.2	0.014 ^†^	37.4 ± 35.9 ^a^
	None	2.1 ± 1.2	2.0 ± 1.1	0.943	1.1 ± 56.9 ^b^
*Culex quinquefasciatus* (Susceptible)	KU #1	1.0 ± 1.1	1.6 ± 1.6	0.527	18.5 ± 69.7 ^c^
	KU #2	2.3 ± 2.3	2.6 ± 1.5	0.587	14.0 ± 50.3 ^c^
	KU #3	4.1 ± 1.7	7.2 ± 5.2	0.235	9.0 ± 61.3 ^c^
	KU #4	1.7 ± 1.0	3.4 ± 2.2	0.041 ^†^	29.6 ± 32.4 ^b^
	KU #5	2.0 ± 1.2	1.0 ± 0.7	0.071	−27.0 ± 61.9 ^d^
	KU #6	3.2 ± 2.4	6.8 ± 2.7	0.007 ^†^	37.2 ± 47.6 ^ab^
	BG-lure	1.1 ± 1.1	5.4 ± 2.2	0.007 ^†^	68.3 ± 25.4 ^a^
	None	3.6 ± 2.7	3.7 ± 3.2	1.000	−18.4 ± 47.4 ^d^
*Culex quinquefasciatus* (Resistant)	KU #1	0.4 ± 0.5	0.3 ± 0.5	0.564	−11.1 ± 60.1 ^c^
	KU #2	6.7 ± 3.7	0.9 ± 1.1	0.011	−70.9 ± 44.7 ^d^
	KU #3	3.9 ± 2.8	0.7 ± 0.9	0.012	−71.5 ± 37.5 ^d^
	KU #4	2.7 ± 3.3	1.9 ± 1.7	0.491	10.1 ± 65.4 ^b^
	KU #5	6.2 ± 3.4	1.9 ± 1.3	0.011	−47.4 ± 25.7 ^cd^
	KU #6	1.8 ± 1.7	4.4 ± 2.9	0.017 ^†^	34.3 ± 61.3 ^a^
	BG-lure	1.2 ± 1.4	3.9 ± 3.2	0.028 ^†^	32.2 ± 80.5 ^a^
	None	1.0 ± 1.4	1.1 ± 1.6	0.892	12.2 ± 62.9 ^b^
*Anopheles minimus* (Susceptible)	KU #1	2.2 ± 2.0	3.4 ± 3.4	0.440	11.4 ± 80.0 ^b^
	KU #2	4.3 ± 2.2	3.0 ± 3.7	0.170	−37.1 ± 49.0 ^c^
	KU #3	3.8 ± 3.2	1.8 ± 2.1	0.160	−27.8 ± 64.5 ^c^
	KU #4	2.1 ± 1.2	1.1 ± 1.3	0.233	−36.3 ± 65.2 ^c^
	KU #5	4.3 ± 2.0	1.2 ± 0.7	0.014	−49.5 ± 38.6 ^d^
	KU #6	0.7 ± 1.3	2.6 ± 0.9	0.014 ^†^	74.3 ± 42.0 ^a^
	BG-lure	0.8 ± 1.0	4.6 ± 4.3	0.018 ^†^	66.7 ± 45.8 ^a^
	None	2.4 ± 1.5	3.3 ± 2.8	0.254	11.8 ± 57.0 ^b^

^†^ Significantly more mosquitoes in the treated chamber. From nine assay replicates, 180 mosquitoes total per mosquito strain. Discriminating dose for all KU-lures (0.01 g). Optimal amount of BG-lure (0.05 g) used as positive control. * Wilcoxon signed rank test (*p* < 0.05). ** Percent attraction = (# mosquitoes in treated − untreated)/(# mosquitoes in treated + untreated) × 100. Different letters in columns indicate significant differences between species-specific doses using Kruskal–Wallis *H* test for multiple comparisons (*p* < 0.05).

## Data Availability

The datasets supporting the conclusions of this article are included within the article. Raw data are available from the corresponding author on reasonable request.

## References

[1] Achee N.L., Youngblood L., Bangs M.J., Lavery V., James S. (2015). Considerations for the use of human participants in vector biology research: A tool for investigators and regulators. Vector Borne Zoonotic Dis..

[2] Kilama M., Smith D.L., Hutchinson R., Kigozi R., Yeka A., Lavoy G., Kamya M.R., Staedke S.G., Donnelly M.J., Drakeley C. (2014). Estimating the annual entomological inoculation rate for *Plasmodium falciparum* transmitted by *Anopheles gambiae sl* using three sampling methods in Uganda. Malar. J..

[3] Lima J.B., Rosa-Freitas M.G., Rodovalho C.M., Santos F., Lourenço-de-Oliveira R. (2014). Is there an efficient trap or collection method for sampling *Anopheles darlingi* and other malaria vectors that can describe the essential parameters affecting transmission dynamics as effectively as human landing catches? A review. Mem. Inst. Oswaldo Cruz.

[4] Sithiprasasna R., Jaichapor B., Chanaimongkol S., Khongtak P., Lealsirivattanakul T., Tiang-Trong S., Burkett D.A., Perich M.J., Wirtz R.A., Coleman R.E. (2004). Evaluation of candidate traps as tools for conducting surveillance for *Anopheles* mosquitoes in a malaria-endemic area in western Thailand. J. Med. Entomol..

[5] Muturi E.J., Mwangangi J., Shililu J., Muriu S., Jacob B., Mbogo C.M., John G., Novak R. (2007). Evaluation of four sampling techniques for surveillance of *Culex quinquefasciatus* (Diptera: Culicidae) and other mosquitoes in African rice agroecosystems. J. Med. Entomol..

[6] Hiwat H., Andriessen R., Rijk M.D., Koenraadt C.J.M., Takken W. (2011). Carbon dioxide baited trap catches do not correlate with human landing collections of *Anopheles aquasalis* in Suriname. Mem. Inst. Oswaldo Cruz.

[7] Costantini C., Li S.G., Torre A.D., Sagnon N.F., Coluzzi M., Taylor C.E. (1996). Density, survival and dispersal of *Anopheles gambiae* complex mosquitoes in a West African Sudan savanna village. Med. Vet. Entomol..

[8] Costantini C., Sagnon N.F., della Torre A.L., Diallo M., Brady J., Gibson G., Coluzzi M. (1998). Odor-mediated host preferences of West African mosquitoes, with particular reference to malaria vectors. Am. J. Trop. Med. Hyg..

[9] Geier M., Bosch O.J., Boeckh J. (1999). Ammonia as an attractive component of host odour for the yellow fever mosquito, *Aedes aegypti*. Chem. Senses.

[10] Grant A.J., Wigton B.E., Aghajanian J.G., O’Connell R.J. (1995). Electrophysiological responses of receptor neurons in mosquito maxillary palp sensilla to carbon dioxide. J. Comp. Physiol..

[11] Syed A., Leal W.S. (2008). Mosquitoes smell and avoid the insect repellent DEET. Proc. Natl. Acad. Sci. USA.

[12] Acree F., Turner R.B., Gouck H.K., Beroza M., Smith N. (1968). L-lactic acid: A mosquito attractant isolated from humans. Science.

[13] Smith C.N., Smith N., Gouck H.K., Weidhaas D.E., Gilbert I.H., Mayer M.S., Smittle B.J., Hofbauer A. (1970). L-lactic acid as a factor in the attraction of *Aedes aegypti* (Diptera: Culicidae) to human hosts. Ann. Entomol. Soc. Am..

[14] Hall D.R., Beevor P.S., Cork A., Nesbitt B.F., Vale G.A. (1984). 1-Octen-3-ol. Int. J. Trop. Insect Sci..

[15] Dogan E.B., Rossignol P.A. (1999). An olfactometer for discriminating between attraction, inhibition, and repellency in mosquitoes (Diptera: Culicidae). J. Med. Entomol..

[16] Mayer M.S., James J.D. (1969). Attraction of *Aedes aegypti* (L.): Responses to human arms, carbon dioxide, and air currents in a new type of olfactometer. Bull. Entomol. Res..

[17] Geier M., Sass H., Boeckh J. (1996). A search for components in human body odour that attract females of *Aedes aegypti*. Olfaction in Mosquito-Host Interactions.

[18] Clements A.N. (1999). Chapter 38 Host finding. The Biology of Mosquitoes. Volume 2: Sensory Reception and Behavior.

[19] Kline D.L., Takken W., Wood J.R., Carlson D.A. (1990). Field studies on the potential of butanone, carbon dioxide, honey extract, 1-octen-3-ol, L-lactic acid and phenols as attractants for mosquitoes. Med. Vet. Entomol..

[20] World Health Organization (2013). Guidelines for Efficacy Testing of Spatial Repellents.

[21] World Health Organization (2018). Efficacy-Testing of Traps for Control of *Aedes* Spp. Mosquito Vectors.

[22] Rudolfs W. (1922). Chemotropism of mosquitoes. N. J. Agric. Exp. Stn..

[23] Willis E.R. (1947). The olfactory responses of female mosquitoes. J. Econ. Entomol..

[24] Brown A.W.A., Sarkaria D.S., Thompson R.P. (1951). Studies on the responses of the female *Aëdes mosquito*. Part I.—The search for attractant vapours. Bull. Entomol. Res..

[25] Meza F.C., Roberts J.M., Sobhy I.S., Okumu F.O., Tripet F., Bruce T.J.A. (2020). Behavioural and electrophysiological responses of female *Anopheles gambiae* mosquitoes to volatiles from a mango bait. J. Chem. Ecol..

[26] Grieco J.P., Achee N.L., Sardelis M.R., Chauhan K.R., Roberts D.R. (2005). A novel high-throughput screening system to evaluate the behavioral response of adult mosquitoes to chemicals. J. Am. Mosq. Control Assoc..

[27] Kim D.Y., Leepasert T., Bangs M.J., Chareonviriyaphap T. (2021). Dose-response assay for synthetic mosquito (Diptera: Culicidae) attractant using a high-throughput screening system. Insects.

[28] Yu J.J., Bong L.J., Panthawong A., Chareonviriyaphap T., Neoh K.B. (2020). Repellency and contact irritancy responses of *Aedes aegypti* (Diptera: Culicidae) against deltamethrin and permethrin: A cross-regional comparison. J. Med. Entomol..

[29] Yang L., Norris E.J., Jiang S., Rernier U.R., Linthicum K.J., Bloomquist J.R. (2020). Reduced effectiveness of repellents in a pyrethroid-resistant strain of *Aedes aegypti* (Diptera: Culicidae) and its correlation with olfactory sensitivity. Pest Manag. Sci..

[30] Kongmee M., Prabaripai A., Akratanakul P., Bangs M.J., Chareonviriyaphap T. (2004). Behavioral responses of *Aedes aegypti* (Diptera: Culicidae) exposed to deltamethrin and possible implications for disease control. J. Med. Entomol..

[31] Sathantriphop S., Paeporn P., Supaphathom K. (2006). Detection of insecticides resistance status in *Culex quinquefasciatus* and *Aedes aegypti* to four major groups of insecticides. Trop. Biomed..

[32] Nararak J., Sathantriphop S., Kongmee M., Mahiou-Leddet V., Ollivier E., Manguin S., Chareonviriyaphap T. (2019). Excito-repellent activity of β-caryophyllene oxide against *Aedes aegypti* and *Anopheles minimus*. Acta Trop..

[33] Chen Z., Guo S., Cao J., Pang X., Geng Z., Wang Y., Zhang Z., Du S. (2018). Insecticidal and repellent activity of essential oil from *Amomum villosum Lour.* and its main compounds against two stored-product insects. Int. J. Food Prop..

[34] Afify A., Horlacher B., Roller J., Galizia C.G. (2014). Different repellents for *Aedes aegypti* against blood-feeding and oviposition. PLoS ONE.

[35] Carey A.F., Wang G., Su C.-Y., Zwiebel L.J., Carlson J.R. (2010). Odorant reception in the malaria mosquito *Anopheles gambiae*. Nature.

[36] Kline D.L. (1994). Olfactory attractants for mosquito surveillance and control: 1-octen-3-ol. J. Am. Mosq. Control Assoc..

[37] Van Loon J.J., Smallegange R.C., Bukovinszkine-Kiss G., Jacobs F., De Rijk M., Mukabana W.R., Verhulst N.O., Menger D.J., Takken W. (2015). Mosquito attraction: Crucial role of carbon dioxide in formulation of a five-component blend of human-derived volatiles. J. Chem. Ecol..

[38] Allan S.A., Bernier U.R., Kline D.L. (2006). Attraction of mosquitoes to volatiles associated with blood. J. Vector Ecol..

[39] Aldridge R.L., Britch S.C., Allan S.A., Tsikolia M., Calix L.C., Bernier U.R., Linthicum K.J. (2016). Comparison of volatiles and mosquito capture efficacy for three carbohydrate sources in a yeast-fermentation CO_2_ generator. J. Am. Mosq. Control Assoc..

[40] Mathew N., Ayyanar E., Shanmugavelu S., Muthuswamy K. (2013). Mosquito attractant blends to trap host seeking *Aedes aegypti*. Parasitol. Res..

[41] Bosch O.J., Geier M., Boeckh J. (2000). Contribution of fatty acids to olfactory host finding of female *Aedes aegypti*. Chem. Senses.

[42] Steib B.M., Geier M., Boeckh J. (2001). The effect of lactic acid on odour-related host preference of yellow fever mosquitoes. Chem. Senses.

[43] Kellogg F.E. (1970). Water vapour and carbon dioxide receptors in *Aedes aegypti*. J. Insect Physiol..

[44] Wolff G.H., Riffell J.A. (2018). Olfaction, experience and neural mechanisms underlying mosquito host preference. J. Exp. Biol..

[45] Kusakabe Y., Ikeshoji T. (1990). Comparative attractancy of physical and chemical stimuli to aedine mosquitoes. Med. Entomol. Zool..

[46] McBride C.S., Baier F., Omondi A.B., Spitzer S.A., Lutomiah J., Sang R., Ignell R., Vosshall L.B. (2014). Evolution of mosquito preference for humans linked to an odorant receptor. Nature.

[47] Wilson R.I. (2013). Early olfactory processing in Drosophila: Mechanisms and principles. Annu. Rev. Neurosci..

[48] Sukumaran D. (2016). A review on use of attractants and traps for host seeking *Aedes aegypti* mosquitoes. Indian J. Nat. Prod. Resour..

[49] Henderson B.E., McCrae A.W., Kirya B.G., Ssenkubuge Y., Sempala S.D. (1972). Arbovirus epizootics involving man, mosquitoes and vertebrates at Lunyo, Uganda 1968. Ann. Trop. Med. Parasitol..

[50] Cork A., Park K.C. (1996). Identification of electrophysiologically-active compounds for the malaria mosquito, *Anopheles gambiae*, in human sweat extracts. Med. Vet. Entomol..

[51] Bernier U.R., Kline D.L., Posey K.H., Booth M.M., Yost R.A., Barnard D.R. (2003). Synergistic attraction of *Aedes aegypti* (L.) to binary blends of L-lactic acid and acetone, dichloromethane, or dimethyl disulfide. J. Med. Entomol..

[52] Xie L., Yang W., Liu H., Liu T., Xie Y., Lin F., Zhou G., Zhou X., Wu K., Gu J. (2019). Enhancing attraction of the vector mosquito *Aedes albopictus* by using a novel synthetic odorant blend. Parasites Vectors.

[53] Lefevre T., Gouagna L.C., Dabire K.R., Elguero E., Fontenille D., Costantini C., Thomas F. (2009). Evolutionary lability of odour-mediated host preference by the malaria vector *Anopheles gambiae*. Trop. Med. Int. Health.

[54] Takken W., Kline D.L. (1989). Carbon dioxide and 1-octen-3-ol as mosquito attractants. J. Am. Mosq. Control Assoc..

[55] Verhulst N.O., Mukabana W.R., Takken W., Smallegange R.C. (2011). Human skin microbiota and their volatiles as odour baits for the malaria mosquito *Anopheles gambiae ss*. Entomol. Exp. Appl..

